# Establishment and optimization of a new model organism to study early land plant evolution: Germination, cultivation and oospore variation of *Chara braunii* Gmelin, 1826

**DOI:** 10.3389/fpls.2022.987741

**Published:** 2022-11-10

**Authors:** Anja Holzhausen, Nora Stingl, Sophie Rieth, Christine Kühn, Hendrik Schubert, Stefan Andreas Rensing

**Affiliations:** ^1^ Department of Biology, Plant Cell Biology, University of Marburg, Marburg, Germany; ^2^ Institute for Biosciences, Physiology of Plant Metabolism, University of Rostock, Rostock, Germany; ^3^ Institute for Biosciences, Ecology, University of Rostock, Rostock, Germany; ^4^ BIOSS Centre for Biological Signalling Studies, University of Freiburg, Freiburg, Germany

**Keywords:** land plant evolution, Charophyceae, streptophyte algae, long-term cultivation, gibberellic acid, phytochrome effect, oospores

## Abstract

For studying land plant evolution, the establishment and optimization of model organisms representing streptophytic algae, sister to land plants, is essential. Long-term cultivation experiments with *Chara braunii* S276 were performed over 8 years, since 4 years (Nov. 2018) under constant conditions. Additionally, short-term experiments for optimization of culture conditions were performed with three strains of *C. braunii* (S276, NIES-1604 and Lausiger Teiche, LaT-2708). Germination success after application of sterilization agents, addition of gibberellic acid and under different incubation conditions with respect to pre-treatment, irradiance regime and substrate was investigated in order to develop protocols for generative cultivation of at least unialgal cultures. The resulting cultivation protocols for *C. braunii* S276, allowing maintenance of vegetative as well as generative cultures are presented in detail, including protocols for germination induction and growth of sterilized and unsterilized oospores.

## 1 Introduction

The transition from water to land by plants that occurred at least 500 Ma ago and the associated molecular, cellular and physiological adaptations led to an enormous extant plant diversity (Gerrienne et al., 2016; De Vries and Archibald, 2018; De Vries and De Vries, 2018). Numerous genomes and transcriptomes of plants have recently become available (One Thousand Plant Transcriptomes, 2019; Provart et al., 2021; Miryeganeh et al., 2022), allowing studies of evolutionary patterns and traits across plants. The Phragmoplastophyta comprise the Coleochaetophyceae, Charophyceae, Zygnematophyceae and Embryophyta (land plants). Since various studies suggest that Zygnematophyceae are the closest relatives of land plants (Wickett et al., 2014; Cheng et al., 2019), different genomes and transcriptomes became available, e.g. of the unicellular desmid *Penium margaritaceum* (Jiao et al., 2020), or two species of the subclass Spirogloeophycidae, *Spirogloea muscicola* and *Mesotaenium endlicherianum* (Cheng et al., 2019). The number is expected to increase in future. Within the morphologically complex Characeae, the genome of *Chara braunii* S276 (Nishiyama et al., 2018) and organellar genomes of *Nitellopsis obtusa* (Sleith and Karol, 2021) brought Charophyceae research into the genomics era of algae.

Whilst model organisms are available for various classes of bryophytes (Rensing et al., 2008; Szövényi et al., 2015; Shimamura, 2016; Rensing et al., 2020), ferns (De Vries and De Vries, 2018) or brown algae (Coelho et al., 2012), a model system per se does not exist within streptophytic algae (Hedges, 2002). But, different species have been utilized as model system for specific purposes, such as electrophysiology or developmental studies (Beilby, 2019; Zhou and von Schwartzenberg, 2020). Additionally, most algal culture collections are lacking Charophyceae and there is no axenic strain available at all. The recently sequenced genome of the C. *braunii* strain S276 does enable functional analyses of, e.g., hormone signaling pathways, such as for auxin (Vosolsobĕ et al., 2020; Schmidt, 2021), strigolactones (Delaux et al., 2012), gibberellins (Godlewski and Kwiatkowska, 1980; Kwiatkowska and Godlewski, 1980) or genes involved in stress response (Wang et al., 2021). However, the use of a *Chara* strain, that is adopted to constant environmental and sediment conditions, could allow the comparability of e.g., transcriptomic analyses across laboratory boundaries.

*Chara braunii* Gmelin, 1826 (*sect. Charopsis*), named in memory of A. Braun (Gmelin, 1826), is a cosmopolitan species, being red listed in some regions while dominating in others (Zhákova, 2003; Helcom, 2013; Schubert et al., 2015). This species is mainly distributed in shallow temporary wetlands and other aquatic systems such as lakes, fish ponds, pools or flooded field areas (Raabe, 2017). As species with a short annual life cycle compared to other Charophyceae species, *C. braunii* produces a high amount of oospores within few months. Reproduction could occur generatively by means of oospores, but can also sustain by fragmentation of thalli (Casanova, 2014). For the northern hemisphere, germination windows are in late spring and early autumn from August to September (Franke and Doege, 2016; Raabe, 2017). The high morphological variability within *Chara braunii* resulted in unambiguous opinions in regard to the taxonomic status and led to the description of various varieties and forms (Wood, 1965). Results of morphology and phylogenetic analyses of *C. braunii* reveal the existence of genetic differences in *rbc*L genes between the Hawaiian NIES-1604 and different Japanese *C. braunii* specimens that are separated in two cluster (Kato et al., 2008). In contrast to this, studies of Polish specimens, Japanese specimens and eight clones from worldwide herbaria material have shown relatively small size ranges for length or width (Proctor, 1970; Boszke et al., 2008; Kato et al., 2008).

Culture protocols for Charophyceae, especially for short-term cultivation, have been published and tested a variety of media compositions, characterized by different combinations of substrates and media ranging from aerated or synthetic seawater (Forsberg, 1965b; Wüstenberg et al., 2011), tap water (Ernst, 1917), media made from clay extracts (Imahori and Iwasa, 1965; Proctor, 1967), to completely synthetic media (Anderson, 1958; Wetzel and McGregor, 1968; Andrews et al., 1984). Sediments were taken from lakes/rivers (Smart and Barko, 1984), or as combinations with composts (Okazaki et al., 1984b), boiled peat with boiled sea sand (Kuczewski, 1906) over soil extract and rotten leaves (Proctor, 1967) to combinations of leaf mould with black soil/river sand and lime (Sakayama et al., 2004; Sato et al., 2014). Some of them even claiming axenic growth and germination (Forsberg, 1965a; Forsberg, 1965b; Forsberg, 1965c; Chowdary, 2014) they have been shown to be inapplicable for the establishment of long-term cultures due to extensive growth of epiphytes or decreasing fertility and growth rate over time. Moreover, most of the existing culture protocols for *Chara braunii* rely on organic-rich substrates as leaf mud, natural river sediments or layers of black sand or fertilizer (Sakayama et al., 2004; Zhao et al., 2012; Sato et al., 2014), even provoking overgrowth by epiphytes during long-term cultivation.

Light regimes and underwater spectral distribution, have been identified as one of the main factors for the regulation of growth and developmental processes such as seed/oospore germination (Sokol and Stross, 1992; Stross et al., 1995; Rensing et al., 2016; Küster et al., 2004; Pinnel et al., 2005). The adaptation to light regimes is regulated by photoreceptors, including the three classes, phytochroms, cryptochroms/phototropins and UV-B receptors. Phytochroms, classified in light- labile and light-stabile ones, are one of the major photoreceptors families and regulate on biochemical and physiological level processes such as germination, growth and photosynthesis (Inoue et al., 2016; Léger-Daigle et al., 2022). Structural, phytochroms exhibit both termini, one of cyanobacterial and one of proteobacterial origin (Buchberger and Lamparter, 2015). Evolutionary, the phytochrome signaling pathway is originated in chlorophytes with *Chlamydomonas reinhardtii* as earliest diverging organism having UVR8 orthologs (Han et al., 2019). It is assumed that this evolutionary hallmark evolved by the transition from deep seas into shallow water areas. Within Charophyceae, the existence of phytochrome- mediated systems was long-time hypothesized (Sokol and Stross, 1992). Sequence data of three chlorophytes, one moss, one lycophyte and six flowering plants have verified the presence of 11 orthologs of light signaling genes (Han et al., 2017) although their functional implication and interaction are currently unknown (Han et al., 2019).

This study aims to identify optimal culture conditions for *C. braunii* regarding light regime and substrate. Both, long-term cultivation and short-term experiments varying the factors substrates (compost, sediment, quartz or sea-sand), media and irradiance regimes were carried out and combined with nutritional substrate analyses. Oospores of used strains were analyzed for size and morphological differences. Additionally, germination experiments of *C. braunii* oospores under different light regimes were performed.

## 2 Materials and methods

### 2. 1 Strains

For cultivation and germination, three different *C. braunii* strains were used: *C. braunii* S276, NIES-1604, and *C. braunii* Lausiger Teiche, LaT-2708 (named after the date of sampling, 27.08.2019). In addition to this, oospores of *C. braunii* from Ranstadt (Hesse, Germany) were included in oospore analysis.

The non-axenic Japanese freshwater strain S276 (KU-2549; KU-MACC) (Sato et al., 2014; Kawai et al., 2020) originates from Lake Kasumigaura (Ibaraki, Japan). The algae used here, were obtained from KU-MACC and cultivated over a period of 8 years at the Universities of Freiburg and Marburg. The Hawaiian freshwater strain NIES-1604 is closely related to S276, isolated by M. Ishimoto in 1998, and kept at the Microbial Culture Collection at the National Institute for Environmental Studies (NIES). On-site cultivation conditions for NIES-1604 comprise mSWC-2 or SWCN-2 media at 20°C, and 16-20 µmol photons/(s*m^2^) in a light:dark cycle of 10L:14D (Okazaki et al., 1984a; Kato et al., 2008; Sato et al., 2014). Few specimens of *C. braunii* LaT-2708 from Lausiger Teiche (Bad Schmiedeberg, Saxony-Anhalt, Germany) were sampled in 2019 by H. Schubert. The occurrence was first recorded during monitoring by H. Korsch in 2010 (Korsch, 2010). Specimens of *C. braunii* from Ranstadt (Hesse, Germany) were collected by U. Raabe in August 2016. Oospores were cultivated for post-maturation on window sills in west direction.

### 2.2 Long-term cultivation of strains

**Figure 1:**
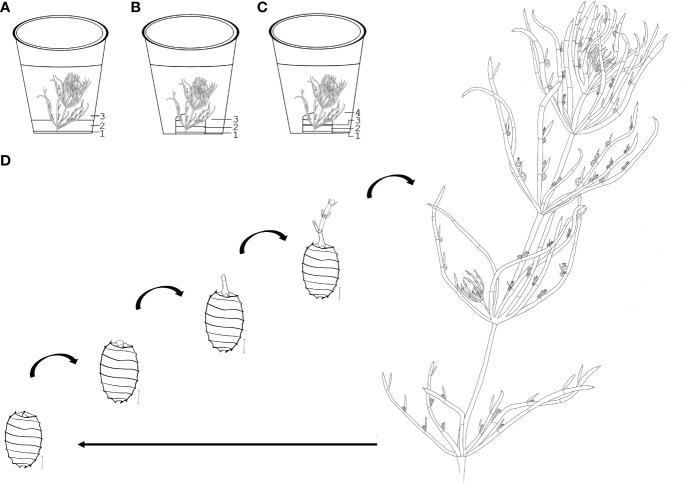
Cultivation methods and morphological units of *C. braunii*. **(A)** direct layering of substrate and quartz sand in cultivation vessels **(B)** layering of substrate and quartz sand in separate autoclaved glass bowl **(C)** layering of substrate, quartz sand and agar in a separate autoclaved glass bowl. **(D)** upper part of a **(C)** braunii thallus (W – whorl, int – internode). 1 – substrate, 2 – quartz sand, 3 – distilled H_2_O or Wüstenberg media, 4 – agar.

**Figure 1** schematically illustrates the cultivation of S276 and NIES-1604 at the University of Marburg. The strains S276 and NIES-1604 were cultivated under relative constant day:night conditions of 22°C:16°C in a light:dark cycle of 16L:8D. Fluorescent lamps (cLED white bi‐phosphor 4000K moisture‐protected LEDs, CLF PlantClimatics GmbH, Germany) provided constant light intensities at a range between 25 and 38µmol photons/(s*m^2^) above the vessels and between 20 and 30 µmol photons/(s*m^2^) below the lids. Manufacturer’s spectral composition of White Light (WL) is given in **Figure S4.1.** These conditions applied, unless explicitly modified for individual experiments. After substrate optimization, culture vessels of different sizes containing double autoclaved layers of compost and quartz sand (0.4-0.8mm in diameter) in a ratio of 10mL compost:100mL quartz per 1L. Compost and quartz sand volumes were calibrated using 15 or 50mL Falcon tubes. In some cases, 0.1g lime per 100mL of quartz were layered between the compost and quartz sand.

Cultivation of *C. braunii* LaT-2708 in modified Wüstenberg medium using sea sand of 0.06 to 0.3mm grain size diameter was conducted first on a windowsill in south direction from August 2019 to February 2020 (August to September, windows were concealed with a white curtain in accordance to Kuczewski, 1906). After the initial experimental period, the cultures were relocated for one month to exclude direct light penetration (laboratory bench), followed by cultivation in east direction from March to May 2020. Temperature and light irradiances were documented using datalogger (MX2202 HOBO Pendant^®^ MX Temperature & Light Data Logger, see **Figure S4.5**).

### 2.3 Short-term experiments

For all short-term experiments, the method of thallus transfer was used. Meaning, the upper thallus part of minimum 2-3 cm was cut off using a sterile tweezers, and transferred into new culture vessels of different sizes (290ml, 340ml, 600ml, 850ml, 1l). For media experiments with S276, mSWC-2 medium (Sakayama et al., 2004) and modified Wüstenberg medium with Ca_3_(PO_4_)_2_ and CoCl_2_ were used. For LaT-2708, Wüstenberg medium with Ca_3_(PO_4_)_2_ was used (Wüstenberg et al., 2011).

Algae were analysed by morphological and developmental parameters such as lengths of thalli, internodes, stipulodes and branchlets plus the presence of contaminations, oogonia, antheridia and oospores. Parameters were tested for normal distribution by Shapiro-Wilk tests using GraphPad Prism 9.3.1 (Graph Pad Software, San Diego, USA). Differences between strains, irradiances, substrates, media and combinations over time were tested using Two-way ANOVA and Tukey’s multiple comparison tests. Significance p-levels were 0.0332 (*), 0.0021 (**), 0.0002 (***) and <0.0001 (****).

#### 2.3.1 Substrate

Algae of the strain S276 were incubated under long day conditions (22°C, 16L:8D, WL) for four weeks using two different composts with five replicates each (n=5 vessels): (1) compost from the Botanical Garden of the University of Marburg (BGUM) and (2) a commercial, certified compost (Gardol^®^, GAR). The same parameters were analyzed as described above. Additionally, nutritional composition of two batches BGUM (2018, 2021) and the commercial compost (2020) as well as the fluid medium were analyzed by the ''Landesbetrieb Hessisches Landeslabor''. Macronutrient analyses were performed using calcium-acetate-lactate extraction, trace elements and heavy metals *via* calcium chloride extraction, cold vapour atomic absorption spectrometry (CV-AAS) and inductively-coupled-plasma mass spectrometry (ICP-MS) and optical emission spectrometry (ICP-EOS). Total nitrogen content, organic carbon and humus were determined *via* dry incineration.

#### 2.3.2 Irradiance

Algae of the strains S276 and NIES-1604 were cultured for four weeks under two different illuminations on BGUM18 substrate in mSWC-2 medium. Three replicates (n=3) of S276 and NIES-1604 were cultivated under WL in a rhythm of 16L:8D and intensities of 70 µm photons/(s*m^2^) (HI), whereas six replicates were incubated under low light intensities of 25-30 µm photons/(s*m^2^) (LI). During this time, samples were frequently evaluated for the increase in biomass, the development of gametangia and contamination. Samples were photographed weekly and measured using Image J.

Short-term cultivation experiments with LaT-2708, started in December 2019. Thalli originating from the long-term cultivation and represent descendants of the south directed culture. Seven replicates (n=7/light condition) each of *C. braunii* LaT-2708 were incubated for six weeks under four different light conditions (measured below the lid): (1) 10-20 µmol photons/(s*m^2^), (2) 60-70 µmol photons/(s*m^2^) (3) 90-100 µmol photons/(s*m^2^), whilst group (4) was cultivated on the windowsill. Incubation temperature was constant at 20°C for (1) – (3). Temperature and light data for the windowsill culture are given in **Figure S4.5**.

### 2.4 Germination experiments

Oospores were sterilized after modified protocols based on Sakayama et al. (2004) and Sato et al. (2014). All used substances and manufacturer’s information are listed in **Table S3**. After sterilization, oospores were plated on LB-, KNOP- and Saboraud-agar plates and incubated at 23°C for testing the sterility success. LB- and KNOP-agar plates were prepared according to Meyberg and Schwartzenberg (2020), Saboraud agar plates consist of 40g/L dextrose, 10g/L peptone and 20g/L agar.

All experiments that were carried out with oospores from *C. braunii* S276, NIES-1604 and LaT-2708 are listed in **Table 3**. Additional information to individual experiments is listed below, manufacturer`s information are given in **Table S3**. Spectral distributions of far-red and white light are given in **Figure S4.1 – S4.4**.

#### 2.4.1 Sterilization protocol

##### 2.4.1.1 Pre-preparations

- double autoclaving of substrate (compost, lake sediment) with an intervening rest period of at least 24 hours and subsequent chilling period of media before use
- double autoclaving of deionized water and germination media modified after Wüstenberg et al., 2011 by using Ca_3_(PO_4_)_2_ and CoCl_2_ for S276 and Ca_3_(PO_4_)_2_ for LaT-2708.
- autoclaved agarose solution (1%, Roth^®^)
- preparing of fresh sodium hypochlorite solution (3%)

##### 2.4.1.2 Oospore treatment and sterilisation

- separation of 15 oospores per 2mL reaction tube in 1mL deionized water
- discarding of supernatant, adding 1mL sodium hypochlorite solution and incubation while shaking for 5minutes (VWR^®^ Tube Rotator and Rotisseries)
- washing 8 times with deionized water for at least 5 minutes (shaking)
- storing in 1 mL deionized water until further use
- control of sterilization by incubation on agar plates

##### 2.4.1.3 Preparation of germination plates

- layering of substrate, oospores and cooled (just before solidification) agarose solution in sterile microtiter plates or glass vessels (we used 50 mL – 290 mL for germination experiments)
- adding germination medium (1 mL for 24 well plates, 3 mL for 6 well plates)
- closing with micropore hypoallergenic papertape (3M)

##### 2.4.1.4 Incubation

- 22°C, 16h light:8h dark, WL < 30 µmol photons/(s*m^2^) until oospores germinate (refilling of evaporated media)

##### 2.4.1.5 After germination

Germlings were transferred after seven to 25 weeks to either mSWC-2 or nutrient agar plates (1% Bold agar with or without compost extract (Nichols and Bold, 1965), for a gradual acclimation incubation from < 30 µmol photons/(s*m^2^) to 50 µmol photons/(s*m^2^) (22°C, 16L:8D, WL) and subsequent transfer of thalli mSWC-2 media with an additional agar layer of 10 mL to suppress nutrient release from compost into the fluid phase.

### 2.4.2 Additional germination experiment information

- gibberellic acid: 1µM (dissolved in distilled water and sterile filtered over 30µm)
- red light pulses: well plates were incubated over a period of 48h hours under red light with dark conditions in between. Red light pulses of 30 minutes were given at incubation start as well as after 4.5 hours and 9 hours; after 48 hours well-plates were constant incubated under white light
- sediment originated from Lausiger Teiche (Bad Schmiedeberg, Saxony-Anhalt, Germany)
- spectral distribution of fluorescent lamps (experiment 5) is given in **Figure S4.4.**

### 2.5 Oospore analysis

Oospores of S276, NIES-1604, LaT-2708 and Ranstadt were analyzed by quantitative and qualitative morphology parameters, defined after Soulié-Märsche and García, 2014. Therefore, oospores were placed on microscope slides with an adhesive surface and documented with a stereomicroscope in lateral, apical and basal view (Leica DM6000 CS). Oospore colour, shape and appendixes such as claws or cages were determined with LI to avoid misinterpretation by photodocumentation. NIES-1604 oospores were pre-treated with sonication for 2minutes at 20°C and 10% Power (Sonorex Super 10P). Parameters, such as number of striae, length, width, fossa width, basal impressions and angle of stria to the longitudinal axis were measured with ImageJ. Statistical data analyses for differences between strains were performed using GraphPad Prism 9.3.1, see paragraph 2.3. (Graph Pad Software, San Diego, USA).

## 3 Results

### 3.1 Long-term cultivation

To find an optimal cultivation protocol that facilitates the reduction of epiphytes, different methods have been tested over the past 4 years (2018-2022). Most of them resulted in contamination and subsequent death of used thalli. These tests are listed in **Table 1**, methods that led to an extremely contaminated state were not further focused on.

**Table 1:** Overview of tested substrate/media compositions on the strains NIES-1604, S276 (NIES-1591, NIES-1593) and Lausiger Teiche (LaT-2708).

No.	Substrate/medium	Strain	Cultivation method to Fig. 1 according to Fig. 1	Irradiance regime	First observable contamination	Success
1	mSWC-2	NIES-1604	1A	green houseHILI	within 1weekwithin 1weekwithin 1week	---
2	mSWC-2 + aquarian water^1^	NIES-1604	1A	HI	after 2weeks	–
3	mSWC-2 + filtered aquarian water^2^	NIES-1604	1A	HI	after 3weeks	–
4	mSWC-2 + ASW	NIES1587	1A	HI	after 2weeks	–
5	mSWC-2	S276	1A	HI	after 6weeks	o
6	mSWC-2	S276	1A	LI	–	+
7	mSWC-2	S276	1B	HI	–	+
8	mSWC-2 + agar^3^	S276	1C	HI	after 4weeks	–
9	mSWC-2 + agar^4^	S276	1C	HI	–	+
10	Wüstenberg^5^	S276	1A	HI	after 3weeks	o
11	Wüstenberg^6^	LaT-2708	1B	windowsill south-side	after 3weeks	o

^1^Aquarian water of the university Marburg (nutrients: Na^2+^ 19.97mg/L; K^+^ 7.06mg/L; Ca^2+^ 32.06mg/L; Mg^2+^ 19.7mg/L; SO_4_^2-^ 61.9mg/L; Cl^-^ 39.5mg/L; F^-^ 0.19mg/L; NO_3_^-^ <0.3mg/L; NO_2_^-^ <0.02mg/L; Umwelthygiene Marburg GmbH & Co KG), ^2^Aquarian water over 30µm filter, ^3^1cm agar layer above quartz/sea sand, ^4^10mL Bold agar above quartz/sea sand, ^5^ Wüstenberg et al., 2011, modified by use of Ca_3_(PO_4_)_2_ and CoCl_2_ x 6 H_2_O, ^6^ Wüstenberg et al., 2011, modified by Ca_3_(PO_4_)_2_. Success is determined by contamination (- no success due to strong contamination and death, o – contamination reduced, + long-term growth under extreme reduced contamination).

In a few vessels, the presence of cyanobacteria was observed after transferring of plants. This could be identified *via* microscopic analyses as e.g. Nostoc sp. or Chlorococcales and confirmed *via* genetic analyses. In most cases, *C. braunii* develop male and unfertilized female gametangia but fertilization seem to be inhibited.

### 3.2 Short-term cultivation

#### 3.2.1 Substrate

Incubation of S276 using two different composts showed significant differences in oogonia formation after one week of incubation (p<0.0001) due to the absence at the beginning and formation within one week using GAR. However, the range of variability for gametangia-specific traits and the formation of new whorls and lateral branches was larger for Gardol^®^ (GAR) than for compost from the Botanical Garden University Marburg (BGUM18, **Table S1**). Using GAR, an increased rate of thallus decay, a reduced and decelerated production of antheridia and oogonia on newly formed lateral branches was observed. In contrast, thalli that were incubated with BGUM18 as substrate, formed and released matured oospores from the main thalli and lateral branches. Seven to eight lateral branches were developed per thalli using BGUM18 and five to eight per thalli on GAR (**Table S1**).

The analysis of the compost types, BGUM from 2018 (BGUM18) and 2021 (BGUM21) and GAR for their nutritional composition (**Table 2**) showed differences in macro- and micronutrient composition. For example, the BGUM18 contained half the phosphorus (P_2_O_5_) and fourfold lower amount of potassium oxide (K_2_O) compared to GAR. Additionally, cations of trace elements such as copper, boron, zinc and cadmium are twofold to fourfold lower in BGUM18 compared to GAR, whereas the total contents, plant unavailable nutrients, were lower in GAR than BGUM18 and BGUM21. Contents of manganese and molybdenum were slightly increased for GAR as compared to BGUM18/21. Between BGUM18 and BGUM21 few differences exist, but seem not to be decisive for cultivation and growth than compared to GAR.

**Table 2:** Results of nutrient analysis of both substrates, the compost from the Botanical Garden of Marburg (BGUM) and Gardol^®^ (GAR).

parameter	BGUM18	BGUM21	GAR
wet bulk density^1^	1287	1230	945
pH	7.3	7.3	7.6
phosphorus (P_2_O_5_)^2^	65	122	137
potassium (K_2_O)^2^	95	111	396
magnesium (Mg)^2^	31	39	46
copper (CAT)^3^	1.63	2.91	3.73
zinc (CAT)^3^	15.2	27.5	63.8
manganese (CAT)^3^	75.5	75.1	87.7
boron (CAT)^3^	1.48	2.87	4.95
molybdenum^3^	0.1	0.06	0.04
TN %	0.27	0.47	0.82
TOC %	3.26	5.14	9.58
C/N ratio	12.07	10.94	11.68
humus content %	5.6	8.8	16.5
plumbum^2^	19.5	24.4	25.7
cadmium^2^	0.19	0.3	0.31
copper (Cu)^3^	17.7	22.1	23.4
chromium^3^	51.2	30.7	9.77
nickel^3^	40.2	30.3	4.82
mercury (Hg)^3^	0.07	0.12	0.08
zinc (Zn)^3^	82.5	108	131

CAT – Cations, plant available nutrients; ^1^ g/L ^2^ mg/100g; ^3^ mg/kg The numbers of both charges lots of BGMU indicate the years 2018 (BGUM18) and 2021 (BGUM21).

#### 3.2.2 Modified Wüstenberg medium

**Figure 2:**
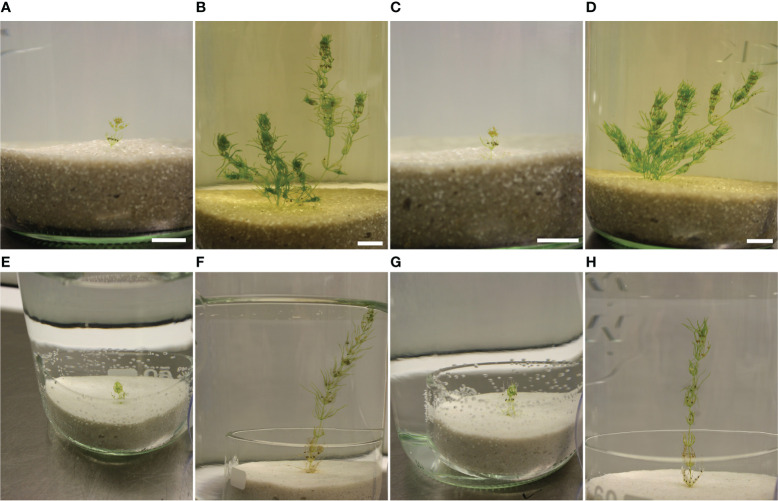
Overview of *C. braunii* S276 cultivation using modified Wüstenberg media or mSWC-2 media. **(A–D)**. Cultivation of *C. braunii* using mSWC-2 media, A+C start of cultivation, B+D after a period of 7 weeks. **(E–H)** Cultivation of *C. braunii* in modified Wüstenberg medium over 7 weeks **(F, H)**.

Cultivation of *C. braunii* S276 using a modified Wüstenberg approach by Ca_3_(PO_4_)_2_ and CoCl_2_ instead of mSWC-2 resulted in a lack of lateral branches (p=0.0138), internode length (p=0.0146), contamination (p<0.0001) and oospore formation and release from the thallus during the experimental phase. The development of lateral branches could not be observed using Wüstenberg medium, whereas branching was regularly observed by cultivation in mSWC-2 medium (**Figure 2**). However, maturation of fertilized oogonia to black oospores occurred faster in mSWC-2 medium compared to Wüstenberg medium (**Table S1**). Moreover, in two out of three vessels with Wüstenberg medium white (not fertilized) oogonia on lower whorls showed signs of contamination and delayed ripening of oospores (**Figure 2**).

#### 3.2.3 Irradiance regime

*C. braunii* NIES-1604 and S276 were incubated under low irradiances (30 µmol photons/(s*m^2^), termed LI) and higher irradiances (70µm photons/(s*m^2^), HI) for four weeks. Generally, differences between strains were higher than between irradiance regimes (**Table S1**). Within the strains, morphologically significant differences and contaminations could only be detected for cultures of NIES-1604 under both, LI and HI in terms of the presence of gametangia. Cultures of S276, however, did not show differences between LI and HI in terms of morphological traits.

Comparing the strains under LI or HI conditions, contamination and the number of oospores on all whorls differ significantly (p<.0001). Compared to S276, a lesser amount of oogonia was produced by NIES-1604 after two (p<.0001) and three weeks (p<.0001) of incubation. The number of lateral branches under HI conditions was higher for S276 (4-8 new lateral branches) than for NIES-1604 (1-3), whereas under LI conditions, both NIES-1604 (1-2) and S276 (1-5) developed fewer lateral branches. No obvious contamination could be detected for S276 during the whole experimental period, whereas all thalli of NIES-1604 were visibly contaminated after one week of incubation with increasing contamination during the further course of the experiment (p<.0001).

Under both conditions, HI and LI, S276 released mature oospores from thalli, whereas only few oospores were released by NIES-1604 under HI conditions. Differences of S276 between HI and LI after 2 weeks resulted from differences of starting material and the non-formation of new oospores within 2 weeks of incubation. Numbers ranged for NIES-1604 under HI from 60 to 103, whereas S276 released under LI 48 to 176 and under HI 120-865 oospores during the whole period of observation (18 months) 18 months. Most oospores of NIES-1604 remained on the thalli or on fragmented whorls.

**Figure 3:**
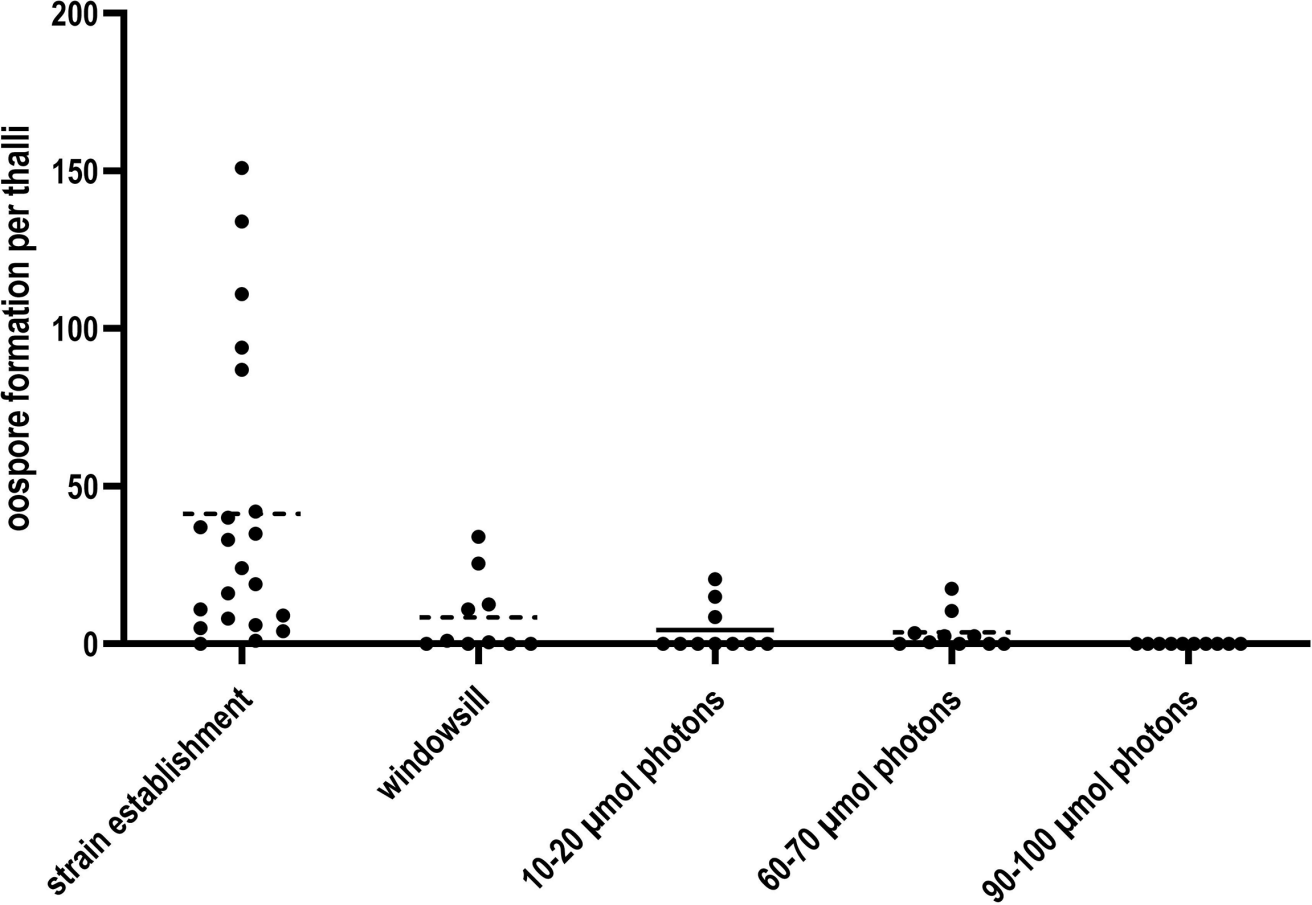
Oospore formation of *C. braunii* LaT-2708 per thalli over 16 weeks strain establishment and the four tested different light irradiances.

Lateral thalli of *C. braunii* LaT-2708 were cultured after strain establishment (16 weeks) for 8 weeks under four different irradiances to test the irradiance effect on cultivation, oospore formation and maturation (**Figure 3**). During this period, only thalli under lowest irradiances produced continuous oospores whereas the number of oospores under intermediate irradiances and on windowsill decreases over time. Only thalli under highest irradiances have completely stopped oospore formation.

In terms of morphology, slight differences among the three irradiances could be achieved by use of fluorescent tubes. The length and width of the last produced internode differed significantly among the irradiance regimes. Shortest and smallest first internodes were obtained under 50 to 60 µmol photons/(s*m^2^), whereas longest and widest first internodes were determined for highest irradiances of 90 to 100 µmol photons/(s*m^2^) (**Table S2**).

### 3.3 Germination experiments

The results of germination assays comparing storage temperatures (4°C or room temperature of approximately 24°C), substrates, the influence of gibberellic acid (GA) and irradiance regimes (white light (WL), red light pulses (RP) or white light with far-red light (WL+FR)) as well as combinations of them are listed in **Table 3**, spectral distributions in **Figures S4.1-S4.4.**

**Table 3:** Results of germination experiments.

Exp. No.	Strain	N (replicates)	T	c(NaOCl), %	c(H_2_O_2_)%	Substrate	Agar	Light	Treatment	Germination rate (%)
**1**	S276	30 (6x5)	RT	10		BGUM18*	1	1		0
		30 (6x5)	RT	10		sed*	1	1		0
		30 (6x5)	RT		10	BGUM18*	1	1		0
		30 (6x5)	RT		10	sed*	1	1		0
		30 (6x5)	RT			BGUM18*	1	1		0
		30 (6x5)	RT			BGUM18	1	1		6.7
		30 (6x5)	RT			sed*	1	1		6.7
		30 (6x5)	RT			sed	1	1		0
**2**	S276	30 (6x5)	RT	10		BGUM18*	1	1		0
		30 (6x5)	RT	10		sed*	1	1		0
		30 (6x5)	RT		10	BGUM18*	1	1		0
		30 (6x5)	RT		10	sed*	1	1		0
		30 (6x5)	RT			BGUM18*	1	1		10
		30 (6x5)	RT			sed	1	1		0
**3**	S276	30 (6x5)	RT	10		BGUM18*	1	1,3		0
		30 (6x5)	RT	10		BGUM18*	1	1,3		0
**3**	S276	30 (6x5)	RT	3		BGUM18*	1	1		0
		30 (6x5)	RT	3		sed*	1	1		0
		30 (6x5)	RT	3		BGUM18*	1	1		3.3
		30 (6x5)	RT	3		sed*	1	1		0
**4**		30 (6x5)	4°C	3		BGUM18*	1	1		3.3
		30 (6x5)	4°C			BGUM18*	1	1		20
		30 (6x5)	4°C	3		BGUM18*		1		0
		30 (6x5)	4°C			BGUM18*		1		0
		30 (6x5)	RT	3		BGUM18*	1	1		0
		30 (6x5)	RT			BGUM18*	1	1		13.3
		30 (6x5)	RT	3		BGUM18*		1		0
		30 (6x5)	RT			BGUM18*		1		0
**5**	S276	30 (6x5)	4°C	3		BGUM18*	1	1		0
		30 (6x5)	4°C			BGUM18*	1	1		6.7
		30 (6x5)	RT	3		BGUM18*	1	1		23.3
		30 (6x5)	RT			BGUM18*	1	1		3.3
		30 (6x5)	4°C	3		BGUM18*	1	1	GA	0
		30 (6x5)	4°C			BGUM18*	1	1	GA	0
		30 (6x5)	RT	3		BGUM18*	1	1	GA	0
		30 (6x5)	RT			BGUM18*	1	1	GA	0
		30 (6x5)	4°C	3		BGUM18*	1	1,2		0
		30 (6x5)	4°C			BGUM18*	1	1,2		0
		30 (6x5)	RT	3		BGUM18*	1	1,2		10
		30 (6x5)	RT			BGUM18*	1	1,2		0
		30 (6x5)	4°C	3		BGUM18*	1	1,2	GA	0
		30 (6x5)	4°C			BGUM18*	1	1,2	GA	6.7
		30 (6x5)	RT	3		BGUM18*	1	1,2	GA	0
		30 (6x5)	RT			BGUM18*	1	1,2	GA	66.7
**6**	S276	30 (6x5)	23°C	5		sed*	2	1		0
	S276	30 (6x5)	23°C	5		BGUM18*	2	1		0
	S276	30 (6x5)	23°C	5		sed	2	1		0
	S276	30 (6x5)	23°C	5		BGUM18	2	1		0
	NIES1604	30 (6x5)	23°C	5		sed*	2	1		0
	NIES1604	30 (6x5)	23°C	5		BGUM18*	2	1		0
	NIES1604	30	23°C	5		sed	2	1		0
	NIES1604	30 (6x5)	23°C	5		BGUM18	2	1		0
	LaT-2708	30 (6x5)	23°C	5		sed*	2	1		56.67
	LaT-2708	30 (6x5)	23°C	5		BGUM18*	2	1		0
	LaT-2708	30 (6x5)	23°C	5		sed	2	1		0
	LaT-2708	30 (6x5)	23°C	5		BGUM18	2	1		0

*Differences in sterilization time and cross-contamination in well-plates. Listed are germination rates of used strains, the number of oospores (n) with replicates in brackets, temperature of storage conditions (T), the concentration of sodium hypochlorite (c(NaOCl, %) or hydrogen peroxide (c(H_2_O_2_, %)), the substrate (BGUM18 – compost from Botanical Garden University Marburg, GAR – compost by Gardol/Germany, sed - sediment from Lausiger Teiche) including the information of sterilization marked with an *, the use of agar (1 – 1% Standard Agarose (Roth, Germany); 2 - 1% LE Agarose (Biozym, Germany) and irradiance regimes (1 – white light, 2 – red light pulses, 3 – combination of far-red and white light) as well as: gibberellic acid (1 µM) for all experiment numbers (exp. no.). All experiments were conducted using Wüstenberg medium. Empty fields in the columns c(NaOCl) and c(H_2_O_2_) meaning non-sterilized oospores. Detailed information are given in section 4.

With respect to pre-treatment, germination rates ranged between 0 and 66.7% for non-sterilized oospores and between 0 and 23.3% for sodium hydrochloride (NaOCl)- treated oospores. Oospores that were treated with hydrogen peroxide do not germinate.

The results show that oospore germination is dependent on the combination of (1) temperature, (2) sterility and (3) the presence of GA and RP. Significant differences can be detected for all assays with (a) low temperatures (4°C) and non-sterilized oospores (ANOVA, p<.0001), (b) room temperatures and a low solution of sodium hypochlorite (3%) (ANOVA,.0003 ≤ p ≤.0079) or (c) room temperatures, non-sterilized oospores and a combination of GA and RP (ANOVA, p<.0001). Using the latter combination, the highest germination rate for non-sterilized oospores (66.7%) was obtained. Lower rates were achieved from oospores stored at room temperatures without further treatment (3.3%). Short-term stratification, long-term desiccation, GA or RP, if applied individually, resulted in no germination at all of *Chara braunii* S276 and NIES-1604 oospores. For NaOCl-treated oospores, the highest germination rate of 23.3% was achieved by oospores stored at room temperature without additional treatments. Germination was also induced on oospores stored at room temperature that were treated with red light pulses (10%) or on oospores stored under cold conditions (4°C, 3.3%).

In germination assays with habitat sediment form Lausiger Teiche (Experiment 6, **Table 3**) as substrate, only oospores from LaT-2708 germinated with a rate of 56.67%. Due to material availability, comparable assays with substrates from Hawaii or Japan could not be carried out.

After germination in 24-well plates under 25-30 µmol photons/(s*m^2^), germlings were transferred either directly into 290 mL vessels using the mSWC-2 method (50-60 µmol photons/(s*m^2^)) or after an intermediate period in Bold-agar with/without compost extract (20-30 µmol photons/(s*m^2^) subsequently transferred to 290 mL vessels under higher irradiances of 50-60 µmol photons/(s*m^2^). Directly transferred germlings either died or showed contaminations after several weeks of incubation and did not develop oospores irrespective of the presence of gametangia. In contrast, germlings that rested for five months in Bold agar with/without compost extract in Wüstenberg medium before being transferred into 290 mL and 600 mL vessels exhibited vegetative growth and developed oospores, except for one individual, after transfer into compost-quartz and distilled water.

**Figure 4:**
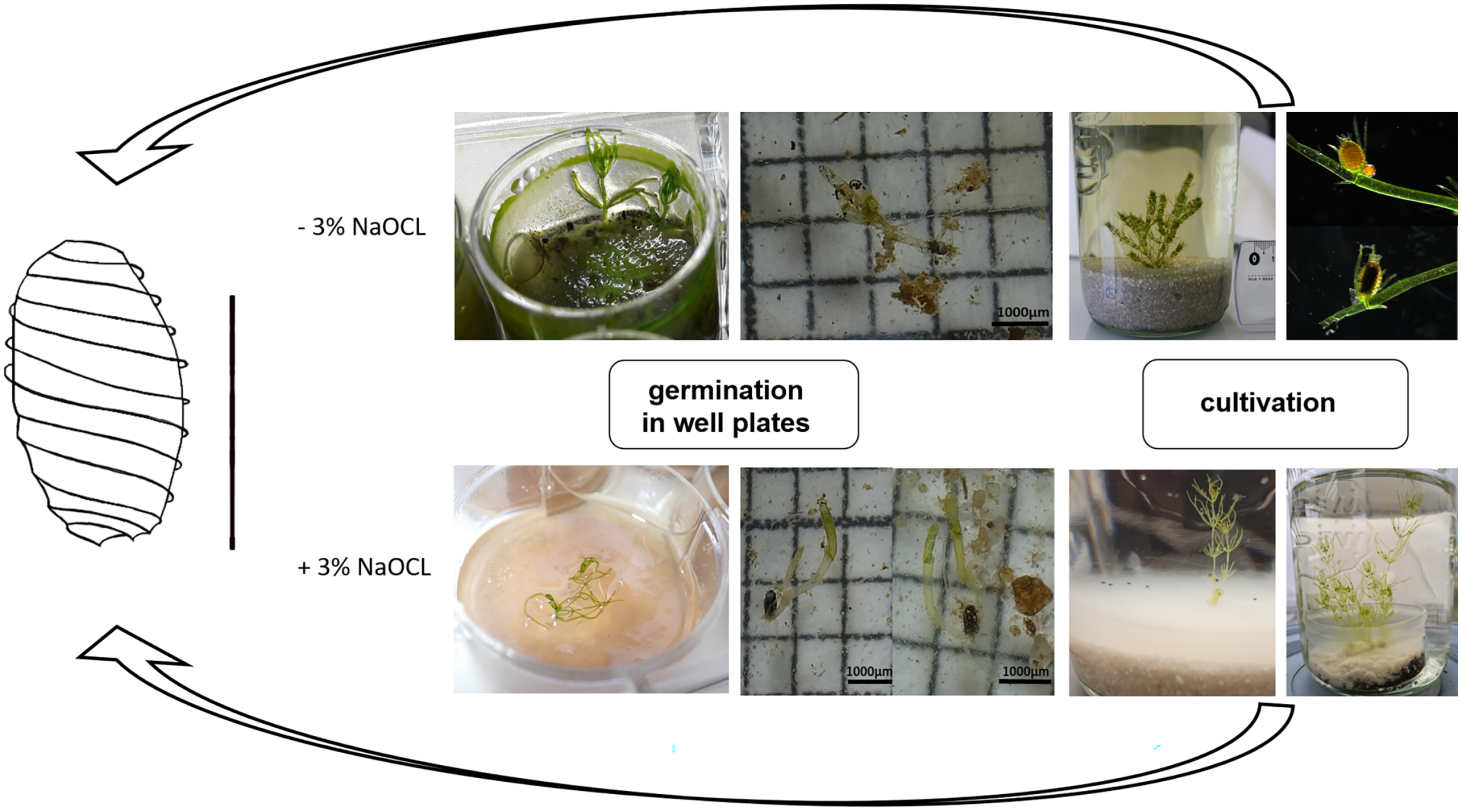
Life cycle cultivation protocol of *C. braunii*. Germlings of *C. braunii* established by germination of non-sterilized (-3% NaOCl; upper panels) or sterilized (+3% NaOCl, lower panels) oospores in well plates. Subsequent cultivation using compost-quartz sand or compost-quartz sand-agar combinations with distilled water led to growth of thalli and reproductive units.

Using this modified protocol, the germination of oospores could successfully be initiated, including the subsequent production of thalli that were able to complete the entire life cycle (**Figure 4**).

**Figure 5:**
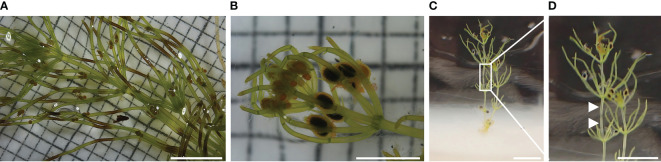
Thalli grown from germinated *C. braunii* S276 oospores after acclimation using Bold agar with or without compost extract. **(A)** thalli grown by germinated, not sterilized oospore with brownish banding pattern on branchlets after acclimation in Bold agar with compost extract, **(B)** thalli grown by germinated, sterilized oospore without brownish banding pattern. **(C)** thalli grown by germinated, sterilized oospore after acclimation in Bold agar without compost extract showing alkaline bands on internodes. **(D)** Detail view of *Chara braunii* internode with alkaline bands (arrows).

Morphological differences were observed between *C. braunii* S276 grown on Bold agar with or without compost extract in gametangia formation and banding pattern (Spear et al., 1969). Germlings in agar with compost extract showed a brownish banding, restricted to branchlets only (**Figure 5A**) and developed no gametangia, whereas those in Bold agar without compost extract developed alkaline bands on internodes and, less pronounced, on branchlets as well (**Figures 5B-D**). Both, antheridia and oogonia were developed on thalli grown in Bold agar. This difference was independent from preceding NaOCl-treatment of the oospores, pointing to a compost effect manifested at the vegetative part.

### 3.4 Interpopulation oospore variation

**Figure 6:**
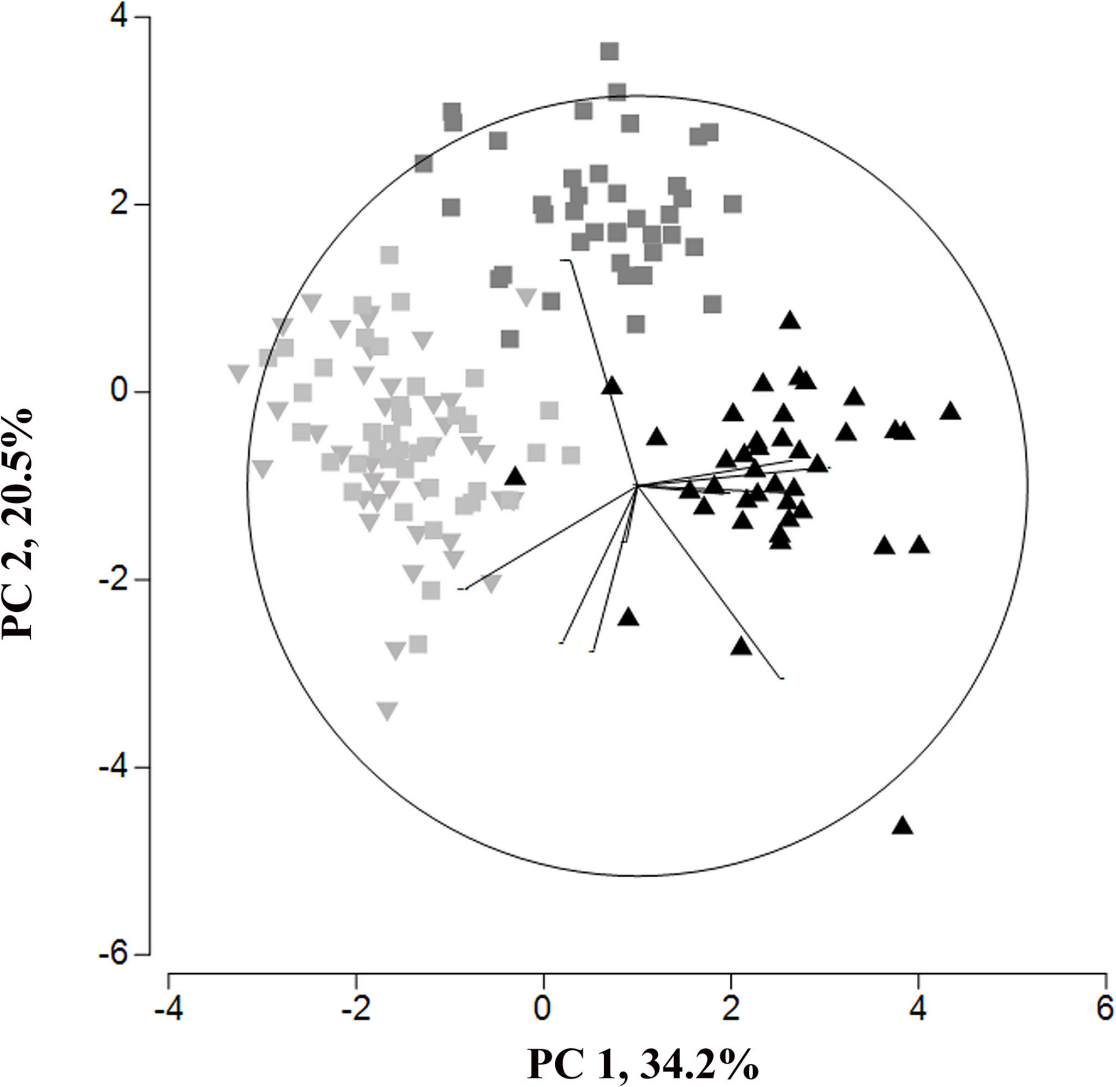
Principal Component analysis of oospore feature from four different *C. braunii* populations (black triangle - S276, Lake Kasumigaura; grey inverted triangle – LaT-2708, Lausiger Teiche; light grey squares – Ranstadt; dark grey squares – NIES-1604, Hawaii).

The principal component analysis of *C. braunii* oospore features of four different populations illustrate a regional separation of S276 oospores from Japan, Hawaii and Germany (**Figure 6**). S276 oospores are longer and broader than oospores from Hawaii (ANOVA, p < 0.0001) or Germany (ANOVA, p < 0.0001) (**Table 4**). Additionally, S276 oospores differ significant from LaT-2708 or Ranstadt by their fossa widths (ANOVA, p<0.0009) or basal impressions (ANOVA, p<0.0001). Oospore features of German sites, LaT-2708 and Ranstadt, overlapped completely.

**Table 4:** Measurements of *Chara braunii* oospore characteristics from Japan (S276), Hawaii (NIES-1604) and Germany (LaT-2708 and Ranstadt).

	n	Striae	Length	Width	Fossae width	Basis impression	Length/width ration
S276	40	6–7 (8-9)± 0.7	459.9-563.7± 23.8 *^1,2,3^	256.5-494.4± 35.6 *^1,2,3^	54.9-81.8± 6.9 *^2,3^	55.8-91.4± 7.6 *^2,3^	1.0-1.9 ± 0.1
NIES-1604	40	5-8 ± 0.7 *^4,5^	421.2-568.1± 31.5 *^4,5^	181.3-264.4± 17.6 *^4,5^	51.6-72.7± 4.9	54.1-79.8± 5.8	1.8-2.5 ± 0.2
LaT-2708	40	6-8 ± 0.6	371.0-558.0± 30.2	200.2-281.5± 22.6	43.4-67.2± 4.6	49.2-89.0± 7.0	1.6-2.3 ± 0.2
Ranstätt	40	6-8 (9) ± 0.7	373.4-498.7± 24.8	202.6-288.7± 22.2	45.1-63.3± 4.4	50.3–74.1± 5.1	1.5-2.3 ± 0.2

All quantitative parameters are listed in micrometre as min-max values with standard deviation. Significant differences between two groups are marked with an asterisk and the number of compared groups (1. S276 - NIES-1604; 2. S276 – LaT-2708; 3. S276 – Ranstadt; 4. NIES-1604 – LaT-2708; 5. NIES-1604 – Ranstadt; 6. LaT-2708 – Ranstadt).

## 4 Discussion

### 4.1 Cultivation

Long-term *in vitro* cultivation is essential for the establishment of model systems applicable in basic as well as applied research. However, maintaining cultures over long periods of time is a challenging task and requires the identification and optimization of parameters that determine plant development, growth, and reproduction, adjusted to the species. In *Arabidopsis thaliana*, long-term cultivation has been linked to age-dependent epigenetic changes such as decreasing proliferation rates, increasing senescence in gene activity, and DNA-methylation (Kwiatkowska et al., 2014). In the moss model *Physcomitrium patens*, cultured since the 1960ties, it could be demonstrated that prolonged vegetative propagation leads to accumulation of deleterious somatic mutations (Haas et al., 2020). Such effects can be avoided by regular sexual reproduction, making the ability to complete the life cycle in cultivation an important asset. However, studies reporting long-term effects appearing under constant cultivation conditions are rare compared to the vast number of short-term acclimation studies.

Extracting the set of factor combinations necessary for the successful *in vitro* cultivation of Charophyceae from the overwhelming complexity of environmental variables is a task requiring a stepwise design followed by multidimensional assays. Results of lab-cultivation approaches, most of them short-term acclimation ones, have been published targeting mainly on the effects of light regime, temperature, substrate, media composition or even the influence of biotic environmental components as, e.g., crustaceans or water slugs (Richter, 1894; Kuczewski, 1906; Ernst, 1917; Karling, 1924; Ding et al., 1991). However, for long term cultivation of model systems factors as, e.g., naturally adhering microorganisms, should be considered as well.

The results presented here showed that long-term maintenance of *C. braunii* S276 by vegetative propagation over 8 years is possible by means of existing protocols, but were accompanied by increasing biofilm-formation and decreased fertility. The addition of organic substrates (BGUM18, BGUM21, **Table 2**) and quarz in combination with deionized water, allowed for constant completion of life cycle without apparent senescence effects. In this context, periodic transfer of thalli to newly prepared culture vessels were shown as option to restrict biofilm formation, although the release of endophytic grown cyanobacteria and algae, e.g. *Coleochaete* sp. (Cimino and Delwiche, 2002) can be triggered.

**Figure 7:**
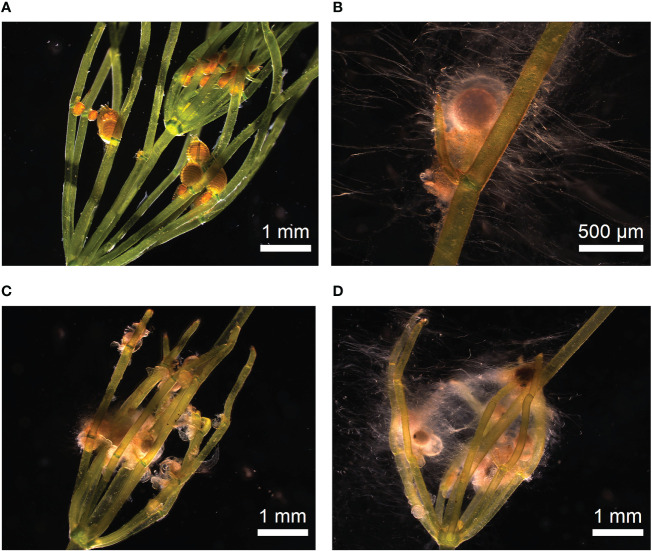
Occurrence of microbes during oogenesis. **(A)** Thalli with fresh antheridia and oogonia after two weeks of incubation. Gametangia showed no visible microbial occurrence. **(B–D)** Microbe occurrence during ripening of gametangia and fertilisation.

For contaminant-reduced cultures, fully synthetic media (with respect to substrate as well as medium) would serve best in theory, because they can serve directly for axenic cultivation of sterilized organisms as well. In Wüstenberg medium, the development of lateral branches was supressed, indicating that the axial bud cell (“Achselursprungszelle”) (Kuczewski, 1906) is formed but did not develop. Addition of compost and use of deionized water as liquid component of the culture overcame this problem; under such conditions lateral formation was regularly observed. The question to what extent the two components – addition of compost and withdrawing nutrients from the liquid phase - contributed to the initialization of lateral development must be investigated in forthcoming long term experiments. Simultaneously, an increased occurrence of microorganisms (fungi), especially on female gametangia, was observable (**Figure 7**). Different associated fungi’s have been identified as endo- and epiphytes (Kataržytė et al., 2017). Detailed interactions and effects on fructification as well as underling gene metabolisms have been proven in further studies.

Compost, natural material resulting from aerobic transformation of organic material by microorganism, is characterized by different composition depending on the raw material and used for increase of soil fertility or plant nutrition. In Germany, quality assurance and certification are implemented by the RAL-quality label (RAL-GZ 251) through the Federal Compost Quality Association. The reproducibility of cultivation results in Charophyceae studies are often restricted by use of composts with not fully defined components (Imahori and Iwasa, 1965; Smart and Barko, 1984; Sakayama et al., 2004; Sato et al., 2014). On the other hand, withdrawing nutrients from the liquid phase consequently requires full-scale nutrition *via* rhizoids including trace elements. In order to develop a completely synthetic medium optimized concentrations of essential elements must be identified in respect to bioavailable forms and chemical reactions. In our approaches and in agreement to observation in natural habitats (Schubert et al., 2018), higher concentrations of available phosphorous, potassium, copper, zinc and boron (labile forms, CAT) cause an increase in phytoplankton and microbes (Cotner and Biddanda, 2002; Gross et al., 2007; Talling, 2010). Simultaneously, decreased potassium concentrations are directly linked with photosynthesis (Tränkner et al., 2018). A chlorophyll decrease is described for *C. zeylanica* (Anderson, 1958). Furthermore, interplays of e.g. sulphate, chloride, potassium and boron’s by increased solubility of potassium-boron’s through higher chloride or sulphate concentrations could effect chemical nutrient cycles but details about interplays are not known so far (Li et al., 2020). For the green alga *Chlorella vulgaris* inhibitory effects of increased boron concentration have been shown including activation of defense mechanisms (Chen et al., 2019), which are linked to increased DNA-methylation and microRNA (miRNA) expression (Wei et al., 2009; Zemach and Zilberman, 2010; Mahbub et al., 2020).

Besides substrate and medium, irradiance is a main factor for autotrophs and several acclimation studies on Charophyceae have demonstrated irradiance effects on oospore lengths, presence of cortication, gametangia development and length growth (Richter, 1894; Karling, 1924; Maszewski, 1980; Sato et al., 2014). However, the variability of this abiotic factor regarding, e.g. spectral distribution, intensity, rhythm, or even angle of incidence, could also hinder the comparability of transcriptomic analyses of e.g., stress or hormonal responses by changes in expression of light complex harvesting genes (Han et al., 2019). Our long-term and short-term studies obtained from *C. braunii* S276 and NIES-1604 under vertical (from above the vessels) WL illumination in LI and HI conditions revealed that differences in morphological and cultural traits are higher between strains than between intensities. Under both conditions, *C. braunii* S276 showed a release of oospores but no visible contamination. Additionally, under HI conditions, a higher biomass production by lateral branches was observed. In contrast to the experiments with Wüstenberg media, lateral branches were formed by both strains under LI and HI conditions, with a reduced number under LI conditions. The impact of changing light conditions on branching remains inconclusive for these experiments but appears less essential than the nutrient-driven lack of formation of lateral branches in Wüstenberg medium. Consequently, the protocol developed can be considered to serve sufficiently uniform material for further studies. Cultivation in deionized water in combination with nutrition at irradiances of 25-40 µmol photons/(s*m2) and 22°C resulted not only at high growth rates (approximately four to nine new whorls) and regular branching, but also the formation and release of oospores. Temperature has been shown an essential factor for fertilization (Croy, 1979). In our studies at cultivation temperatures of 16°C, C. braunii produced both, male and female gametangia, but no fertilization occurred. A suppression of oogonial primordium development at irradiances above 10 µmol photons/(s*m2) as reported in a previous study of Sato et al. (2014) was not observed. The reason for this remains unclear, but given the large differences between the emission characteristics of fluorescence tubes, an effect of spectral composition is a likely candidate, highlighting the importance of following the protocol also in details as, e.g. brand and type of illumination sources.

In contrast, the angle of incidence on windowsill cultures is subject to seasonal change. Variability in cardinal-directed orientation of *Chara* cultures in accordance with natural environmental conditions in aquatic systems is intended to simulate the most natural solar gradient possible for *in vitro* cultures. These results are partly comparable to what could be shown for *Chara fragilis* by Karling (1924). However, the number of oospores was smaller than in experiments with *C. braunii* S276 under constant WL conditions. Cultures that were collected at the beginning of September and cultivated on east-south windowsills died, probably due to the insufficient light intensity necessary for thalli regeneration. Our results have proven that mimicking natural variability in irradiance is not a prerequisite for successful long-term cultivation. Moreover, in contrast to windowsill-cultivation the use of artificial light sources at constant irradiance regimes as done here can serve for season-independent growth and reproduction, making standardized material available throughout the year.

### 4.2 Germination

In order to establish axenic cultures, sterilized plant material must be achieved. Except for the presence of endobacteria, this can be done best by germination of sterilized oospores, which are very resistant to harsh environmental conditions. In addition, controlled induction of germination is a prerequisite for establishing sexually reproducing cultures to prevent depression effects of long-term cultivations as outlined above. Germination of seeds and spores is known to be influenced by abiotic factors such as temperature, light availability, salinity and nutrient conditions, which needs to be investigated the same way as outlined above for vegetative growth. Although theoretical models for breaking the dormancy and inducing germination, such as the hypothetical model for physiological dormancy of Hilhorst (Hilhorst, 1998), exist, the actual application to Charophyceae oospores under *in vitro* conditions is time-consuming and non-trivial. Studies on influential factors such as thermal stratification (Forsberg, 1965c; Forsberg, 1966; Sokol and Stross, 1986; Casanova and Brock, 1996; Stross, 1989), redox potential changes (Proctor, 1967; Bonis et al., 1995; Casanova and Brock, 1996), organic material (Zenker, 2003; Holzhausen et al., 2017), growth inhibitors (Sabbatini et al., 1987), or light regimes (Forsberg, 1965c; Takatori and Imahori, 1971; Sokol and Stross, 1992; Holzhausen et al., 2017) have been conducted for several *Chara*, *Tolypella* and *Nitella* species, demonstrating a large degree of species specificity.

Our results, obtained from non-treated and treated oospores of *C. braunii* S276, support the theory of genetically fixed germination programmes. Stratification of *C. braunii* S276 oospores inhibited germination, whereas storage at room temperature of approximately 22°C ( ± 2°C) promoted germination. Red light stimuli combined with gibberellic acid were first described by Sederias and Colman (2007) to induce germination in *C. vulgaris* and also affected germination of *C. braunii* S276 in our experiments. In contrast to *C. vulgaris*, the effect of red light on germination of *C. braunii* could only be detected for oospores stored at room temperature and not for stratified oospores. Germination induction of seeds *via* red light treatment is known for many plant species, e.g. for European forest seeds (Kolodziejek et al., 2017). These results could be linked with the natural origin of the wildtype material of *C. braunii* S276 from the region of Zonobiom V with temperature-dependent seasons and short cool winters (Pott, 2005). Germination assays under LI conditions combined with far-red light failed in this study. Our results show, that *C. braunii* for sure is not a negatively photoblastic species, which would only germinate in darkness. Moreover, the observed stimulating effect of red light pulses suggest the potential involvement of a phytochrome-like receptor system in germination induction.

Germination rates of oospores under WL cLED’s were lower than those obtained by combination of red light, gibberellic acid and BGUM18 substrate, but approximately the same as induced by the red light stimulus as individual factor. These results fully coincide with studies of Takatori and Imahori on *C. delicatula* (1971). The spectra of the cLEDs used here correspond, except for the short-wavelength edge, well with underwater spectra in shallow coastal lagoons (Sagert and Schubert, 1999).

Since germination of oospores could not be induced *via* treatment with gibberellic acid alone, but seemed to increase the promoting effect of red light, combination effects of other factors are not unlikely and offer a fascinating field for further investigations by, e.g. transcriptomic analyses of unravelling the germination process in detail.

The link of light and nitrogen availability affecting germination of seeds is not fully understood but could be demonstrated to affect germination of several plant species (Hilhorst and Carssen, 2000; Kolodziejek et al., 2017) as well as Charophyceae (Rodrigo et al., 2007). In our study, no dedicated experiment was performed but slight variations in C:N ratios between the different types of compost used (12:1 for BGUM18, 10.9:1 for BGUM21 and 11.7:1 for GAR) could be at least part of the explanation, why successful germination occurred only with BGUM18, having the highest C:N ratio.

### 4.3 Interpopulation oospore variation

The results of analyzing the oospore variation among four *C. braunii* populations have shown the significant separation of S276 oospores from NIES-1604 as well as the German strains LaT-2708 and Ranstadt on the basis of length, width, fossa width and basal impression. Additonally, oospores of NIES-1604 differ in length and width. Compared to existing data, oospores of S276 and NIES-1604 correspond to the given ranges (Proctor, 1970; Haas, 1994; Krause, 1997; Boszke et al., 2008). Oospores of the German sites, Lausiger Teiche and Ranstadt are shorter and smaller compared to literature data and the remaining strains. Between both German strains, no differences could be detected which underpin the results of regional studies in India, Poland or Sweden with relative uniform *C. braunii* oospores. In regard to recent phylogenetic studies, the results of oospore studies could confirm the results of genetic entities by Kato et al., 2008 by morphological differences between S276 and NIES-1604. Additionally, German strains differ from both, the Hawaiian and Japanese strain and indicate the existence of an additional entity by regional separation based on morphological data. Nonetheless, within German sites and shorter regional boundaries, no differences could be detected and imply the same entity.

## 5 Conclusion

In summary, the results showed a successfully optimized cultivation and germination protocol for *C. braunii*, allowing for stable long-term maintenance of cultures with generative reproduction and able to provide sterile material. In any case the protocol presented here offers a unique opportunity for transcriptomic studies with uniform material and to study the potential microbial effects on the ontogenesis of Charophyceae, whose existence have been hypothesized by Holzhausen, 2016 already. Nonetheless, these studies also suggest that more research is needed in the fascinating field of Charophyceae cultivation and germination, e.g. by optimizing of nutritional media or conditions.

## Data availability statement

The original contributions presented in the study are included in the article/**Supplementary Material**, further inquiries can be directed to the corresponding author.

## Author contributions

Conceptualization – SAR, HS, AH; methodology – AH, CK, SR, NS; validation - AH, CK, SR, NS; formal analysis - AH, CK, SR, NS; investigation - AH, CK, SR, NS; resources – SAR, AH, HS; original draft preparation - AH; review and editing – SAR, HS; visualization - AH; supervision - AH, SAR; funding acquisition – SAR, HS. All authors contributed to the article and approved the submitted version.

## Funding

This research was funded by DFG (CharMod; RE 1697/16-1; Schu983/23-1). The project was associated with MAdLand (http://madland.science, DFG priority program 2237, RE 1697/19-1). Open Access funding provided by the Open Access Publication Fund of Philipps-Universität Marburg with support of the Deutsche Forschungsgemeinschaft (DFG, German Research Foundation).

## Acknowledgments

The authors are thankful for the constructive suggestions of two reviewers, Janine Fürst-Janssen and Eduardo Flores-Sandoval. Thanks are due to the “Hessische Landeslabor”, Jennifer Löber and Fabian Jacobi for preparation of compost and media analyses. Marco Göttig, Evelyn Vollmeister, Fabian Haas (University Marburg) and Susanne Thuemecke provided support for students, data analysis and imaging. Uwe Raabe provided plant material (Ranstadt) and was involved in species discussions; Arne Schoor, Christian Porsche and Birgit Munzert (University Rostock) provided technical support in cultivation setup and spectrophotometric analyses, which is gratefully acknowledged by the authors.

## Conflict of interest

The authors declare that the research was conducted in the absence of any commercial or financial relationships that could be construed as a potential conflict of interest.

## Publisher’s note

All claims expressed in this article are solely those of the authors and do not necessarily represent those of their affiliated organizations, or those of the publisher, the editors and the reviewers. Any product that may be evaluated in this article, or claim that may be made by its manufacturer, is not guaranteed or endorsed by the publisher.

## Supplementary material

Supplementary Figure 4 Spectral distribution of light sources. (1). cLED white bi‐phosphor 4000K moisture‐protected LEDs, PlantClimatics GmbH, (2) cLED far-red, PlantCLimatics GmbH, (3) fluorescent lamps (Signify GmbH (Philips)), (4) fluorescent lamps Phillips TLD 36W/950 (Signify GmbH (Philips)), (5) windowsill culture, data were obtained by datalogger (MX2202 HOBO Pendant^®^ MX Temperature & Light Data Logger).
