# Supplementary figures and images for: Establishment and optimization of a new model organism to study early land plant evolution: Germination, cultivation and oospore variation of Chara braunii Gmelin, 1826
Source: Front Plant Sci. 2022 Nov 10;13:987741. doi: 10.3389/fpls.2022.987741 (PMC9691404; doi:10.3389/fpls.2022.987741)

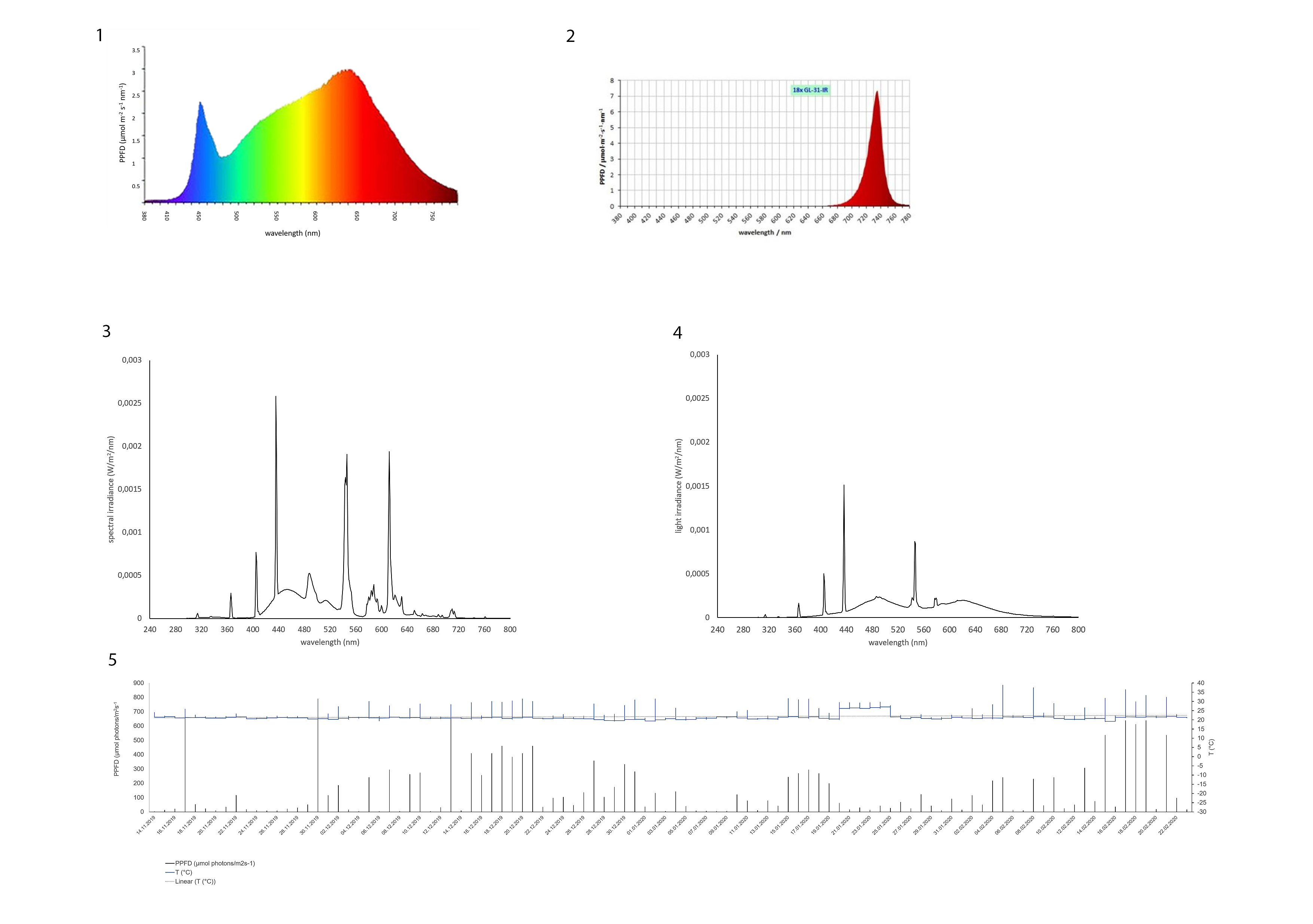

Supplement: Supplementary Figure 4 — Spectral distribution of light sources. (1). cLED white bi‐phosphor 4000K moisture‐protected LEDs, PlantClimatics GmbH, (2) cLED far-red, PlantCLimatics GmbH, (3) fluorescent lamps (Signify GmbH (Philips)), (4) fluorescent lamps Phillips TLD 36W/950 (Signify GmbH (Philips)), (5) windowsill culture, data were obtained by datalogger (MX2202 HOBO Pendant® MX Temperature & Light Data Logger). [file Image_1.png]
